# Exercise-related changes in depressive symptoms and their association with anterior cingulate cortical thickness

**DOI:** 10.1016/j.ijchp.2026.100712

**Published:** 2026-08-08

**Authors:** Qi Xia, Qing Yu, Yuchen Li, Yuanpeng You, Xiaoqi Mo, Zijing Zhang, Chris Baeken, Luqing Wei, Guo-Rong Wu

**Affiliations:** a Key Laboratory of Cognition and Personality, Faculty of Psychology, Southwest University, Chongqing, 400715, China; b Faculty of Medicine and Health Sciences, Department of Head and Skin, Ghent Experimental Psychiatry (GHEP) lab, Ghent University, Ghent, 9000, Belgium; c Eindhoven University of Technology, Department of Electrical Engineering, Eindhoven, the Netherlands; d Department of Psychiatry, NEUR Research Group, Vrije Universiteit Brussel (VUB), Universitair Ziekenhuis Brussel (UZ Brussel), Laarbeeklaan 101, 1090, Brussels, Belgium; e Department of Psychiatry, the First Affiliated Hospital of Chongqing Medical University, key Laboratory of Major Brain Disease and Aging Research (Ministry of Education), Psychiatric Center of Chongqing Medical University the First Affiliated Hospital Chongqing, China

**Keywords:** Aerobic exercise, Anterior cingulate gyrus, Depressive symptoms, Serotonin, Glutamate

## Abstract

Aerobic exercise is associated with reduced depressive symptoms, but the neurobiological processes supporting this effect remain incompletely understood. We investigated whether short-term aerobic exercise reduces depressive symptoms in healthy young adults and whether such reduction is related to cortical structural change and neurotransmitter-receptor organization. Forty-eight participants completed a two-week aerobic running intervention and a two-week control period, with depressive symptoms and structural MRI assessed at baseline, week 2 and week 4. Aerobic exercise significantly reduced depressive symptoms relative to the control period. Across individuals, greater symptom reduction was associated with larger increases in anterior cingulate cortical thickness, an association observed only during the active intervention. The cortical map of this symptom–thickness association showed selective spatial correspondence with normative distributions of serotonergic 5-HT1A, 5-HT1B, 5-HT2A and 5-HT4 receptors, and the glutamatergic mGluR5 receptor, and this correspondence was observed only during the exercise period. These results indicate that short-term aerobic exercise is associated with changes in depressive symptoms and anterior cingulate structure. They also provide a neurobiologically informed context for understanding these exercise-related changes.

## Introduction

Depressive symptoms occur across both clinical and nonclinical populations and are associated with impaired functioning and an increased risk of developing major depressive disorder (MDD). MDD is a prevalent and debilitating mental health condition characterized by high heterogeneity, incidence, and recurrence (Malhi & Mann, 2018). Although pharmacotherapy and psychotherapy have demonstrated efficacy, psychotherapy is often constrained by high costs and modest efficacy, while pharmacotherapy carries risks of adverse effects, poor response, and potential relapse (Cuijpers et al., 2021; Lam et al., 2024). Individuals with mild to subclinical depressive symptoms may also delay or avoid seeking professional support because of stigma, financial constraints, or concerns about medication side effects (Kessler, 2012; Thornicroft et al., 2017). These challenges underscore the need for accessible, low-cost, and easily implemented approaches to reducing depressive symptoms across clinical and nonclinical populations.

Physical exercise has increasingly emerged as a promising intervention for improving mood and reducing depressive symptoms. A growing body of evidence indicates that exercise is associated with reductions in depressive symptoms and may complement conventional treatments in clinical populations (Noetel et al., 2024). For example, a 12-week multimodal exercise program significantly decreased Beck Depression Inventory-II (BDI-II) scores in adolescents with depressive symptoms (Nasstasia et al., 2019). Exercise has been incorporated into clinical guidelines for depression management (Chen et al., 2022; Noetel et al., 2024). Given its accessibility, low cost, and minimal side effects, exercise represents a feasible and scalable strategy for supporting mental health. However, the neurobiological processes underlying exercise-related changes in depressive symptoms remain incompletely understood.

One potential mechanism involves exercise-induced structural brain plasticity. Structural alterations in the hippocampus, anterior cingulate cortex (ACC), and prefrontal cortex have been reported in individuals with depression, whereas exercise has been associated with structural plasticity in these regions (Erickson et al., 2011; Floel et al., 2010; Koolschijn et al., 2009; McEwen et al., 2023; Schmaal et al., 2016; Zhao et al., 2014). Meta-analytic evidence further suggests that structural changes in the hippocampus, ACC, and prefrontal cortex may contribute to exercise-related reductions in depressive symptom (Gujral et al., 2017). Exercise also modulates multiple neurotransmitter systems critical for affective and cognitive processes, including serotonin, dopamine, glutamate (Glu) and Gamma-aminobutyric acid (GABA) (Ando et al., 2024; Maddock et al., 2016; McGregor et al., 2020; Pahlavani, 2024; Zimmer et al., 2016). Together, these findings suggest that exercise-related changes in depressive symptoms may involve interacting structural and neurochemical processes (Kim et al., 2025; Pahlavani, 2024; Ross et al., 2023; Young, 2007). Nevertheless, it remains unclear how neurotransmitter receptor organization relates to exercise-associated cortical structural changes and individual differences in depressive symptom change.

The present study examined changes in depressive symptoms following a two-week running intervention in healthy young adults. We further investigated whether individual differences in symptom change were associated with changes in cortical thickness within regions implicated in mood regulation. Finally, using the JuSpace toolbox, we test whether the spatial pattern of exercise-related symptom–thickness associations align with normative cortical distributions of major neurotransmitter receptors and transporters.

## Materials and methods

### Participants

**Fig. 1:**
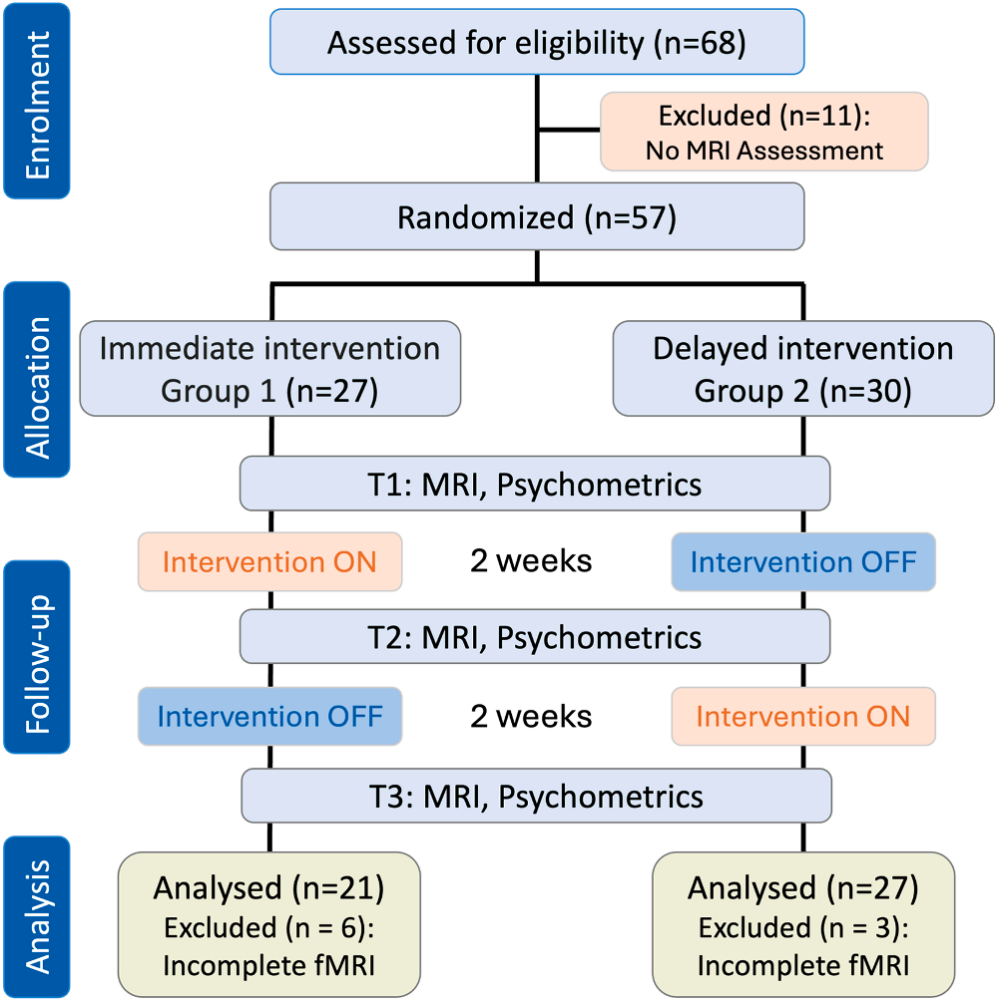
CONSORT diagram depicting participant flow in the crossover intervention study. Participants were randomized to immediate (Group 1) or delayed (Group 2) aerobic exercise intervention. MRI and psychometric assessments were conducted at baseline (T1), midpoint (T2), and follow-up (T3). Group 1 underwent intervention between T1 and T2, while Group 2 crossed over to the intervention period after T2.

All data used were publicly available from the OpenNeuro database (https://openneuro.org/datasets/ds003799/versions/1.0.0), and 68 healthy participants—primarily college students—aged 19 to 33 years (M = 23.00, SD = 2.82) were included. Eleven participants missed some MRI assessment sessions, resulting in a final sample of 57 individuals who consented to and initiated in both the running intervention and MRI assessments (see Fig. 1). Among them, 48 participants (27 women) completed all three MRI scans and psychometric evaluations and fully adhered to the intervention protocol. All participants were right-handed (with the exception of one ambidextrous individual) and reported no history of psychiatric disorders. The sample predominantly consisted of individuals with low physical activity levels, with an average weekly exercise duration of approximately 0.5 h (M = 0.53, SD = 1.2). Participants were screened using a safety questionnaire assessing potential exclusion criteria (e.g., metallic implants, cardiac pacemaker, epilepsy, tattoos, piercings), in compliance with MRI safety standards.

Written informed consent was obtained from all participants. The use of this open-access dataset (OpenNeuro: ds003799) was conducted in compliance with the data sharing policies of the OpenNeuro platform and under the ethical approval originally granted by the ethics committee of the University of Graz for the primary data collection. As this study involved secondary analysis of anonymized, publicly available data, no additional ethical approval was required.

### Running intervention design

Forty-eight participants were randomly assigned to two groups with different intervention timings: Group 1 (n = 21, 11 females) received the running intervention during Weeks 1–2, while Group 2 (n = 27, 16 females) received the same intervention during Weeks 3–4. The structured running program lasted two weeks for each group. Assessments were conducted at three time points: baseline (T1), two weeks (T2), and four weeks (T3). At each time point, participants underwent MRI scans and completed the Center for Epidemiologic Studies Depression Scale (CES-D) to evaluate depressive symptoms (Radloff, 1977).

The running route is situated within a forested recreational area, extending approximately 5 km with an elevation difference of around 100 m Participants completed seven running sessions over two weeks. Each session lasted 50–60 min, including warm-up and cool-down exercises. Runs were conducted in groups, with at least one supervisor present to ensure adherence and safety. Further details on intervention procedures can be found in previous studies (Fink et al., 2021).

### Psychometric tests

Depressive symptoms were assessed using the German version of the CES-D. The CES-D is a brief self-rating scale commonly used to evaluate depressive symptoms within the general population (Radloff, 1977). It consists of 20 items, grouped into four factors related to depressive symptoms: positive emotions, negative emotions, physical symptoms, and interpersonal problems (Shafer, 2006). Participants rated how frequently they experienced each condition over the past week on a four-point scale, ranging from “infrequent” (0) to “the most time” (3). According to the scale’s guidelines, a score of 22 or higher is considered indicative of a level of depressive symptomatology that warrants further clinical evaluation.

### MRI data acquisition and processing

MRI scanning was scheduled at three time points (T1, T2, and T3) using a 3.0 T Siemens Skyra scanner equipped with a 32-channel head coil. T1-weighted structural images were obtained using a 3D MPRAGE sequence (TR = 2200 ms, TE = 2.18 ms, flip angle = 8°, voxel size = 0.88 mm isotropic, matrix = 256 × 256, bandwidth = 210 Hz/Px; total acquisition time: 5 min 51 s) (Fink et al., 2021).

T1-weighted (T1w) images from three timepoints (T1, T2, T3) were processed independently using fMRIPrep, with cortical surface reconstruction performed via FreeSurfer’s *recon-all* (version 6.0.1). All reconstructed data underwent thorough visual inspection to ensure the accuracy of registration, skull stripping, segmentation, and cortical surface reconstruction. To improve longitudinal consistency and reduce intra-individual variability, FreeSurfer’s longitudinal pipeline was applied to the recon-all outputs. A subject-specific unbiased template was first generated using *recon-all -base*, providing a stable reference for subsequent timepoint-specific processing. Each timepoint was then reprocessed with *recon-all -long*, leveraging the template’s geometry to refine surface reconstruction and improve cross-timepoint alignment. Cortical thickness maps were subsequently projected onto the *fsaverage* template and smoothed with a 10 mm full-width at half-maximum (FWHM) Gaussian kernel for group-level analysis. This approach enhances sensitivity to subtle cortical changes while ensuring robust and reproducible measurements over time.

### Statistical analyses

**Fig. 2:**
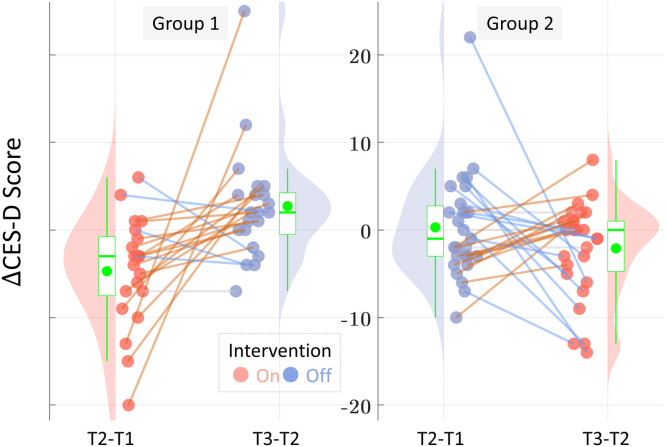
Individual-level changes in ΔCES-D scores during periods with (intervention on) and without (intervention off) aerobic exercise. Red lines indicate a relative increase in CES-D change scores, while blue lines indicate a relative decrease.

To evaluate the effect of aerobic exercise on depressive symptoms over time, we employed a linear mixed-effects model implemented with the ***lmerTest*** package in R. The dependent variable was defined as the change in depressive symptom scores ($\Delta CESD=\text{CES}D_{i+1}-\text{CES}D_{i}$), calculated separately for two intervals: T1–T2 and T2–T3. Individual-level changes are shown in Fig. 2. Fixed effects included Intervention (whether participants received the running program during the respective interval: active vs. control), Time (T1–T2 vs. T2–T3), and their interaction (Intervention ×Time). Age and sex were included as covariates. A random intercept for each subject accounted for the repeated measures design. Model significance was determined via Type III ANOVA with Satterthwaite’s approximation for degrees of freedom. Estimated marginal means were calculated using the *emmeans* package. Statistical significance was set at *p* < 0.05.

For MRI analyses, we examined whether percent changes in cortical thickness (CT) were associated with depressive symptom improvement specifically during active intervention periods. Percent change was used ($\%\Delta CT=\left(CT_{i+1}-CT_{i}\right)/CT_{i}$) to account for baseline anatomical variability and to facilitate interpretable, proportional associations. Similarly, CES-D percent change (%ΔCESD) was computed.

We first focused on the active intervention periods—Group1 (T1–T2) and Group2 (T2–T3)—to identify cortical regions where percent change in CT correlated with symptom reduction. Whole-brain vertex-wise regression analyses were conducted using SPM12, with CES-D percent change as the predictor and CT percent change maps as the dependent variable. Age and sex were included as covariates. Multiple comparisons were corrected using random field theory (RFT), applying a cluster-forming threshold of uncorrected vertex-wise p<0.001 and a cluster-level threshold of p<0.05 (Chumbley et al., 2010).

To test whether this relationship was specific to the intervention periods, we then conducted a moderation analysis within the identified cortical regions across all intervals (including both intervention and waitlist phases). A linear mixed-effects model was implemented in R with the following specification:

%ΔCT∼%ΔCESD×Intervention+Time+Age+Sex+(1|Subject).

The interaction term tested whether the strength of the association between CT and CES-D changes differed depending on intervention status. Subject-level random intercepts accounted for repeated measures.

Finally, to explore potential neurochemical mechanisms, we used the JuSpace toolbox to assess the spatial correspondence between the unthresholded *t*-map (reflecting CT-symptom associations during intervention) and normative neurotransmitter receptor maps (including serotonin, dopamine, GABA, and glutamate). Statistical significance was assessed using spin permutation testing, with FDR correction applied across receptor systems.

## Results

### Exercise intervention reduces depressive symptoms

The linear mixed-effects model revealed a significant main effect of Intervention on CES-D change scores (F(1,90)=15.30,p<0.001). Estimated marginal means comparisons confirmed lower CES-D scores during intervention periods compared to non-intervention (t=−3.91,p=0.0003), indicating greater symptom improvement during active aerobic exercise. Despite a significant main effect of Time (F(1,90)=3.99,p=0.049), CES-D change was smaller during T1–T2 than T2–T3, but this difference did not reach significance in pairwise comparisons (t=−2.00,p=0.052). The Intervention × Time interaction was not significant (F(1,90)=0.01,p=0.93).

### The relationship between changes in CES-D scores and cortical thickness

**Fig. 3:**
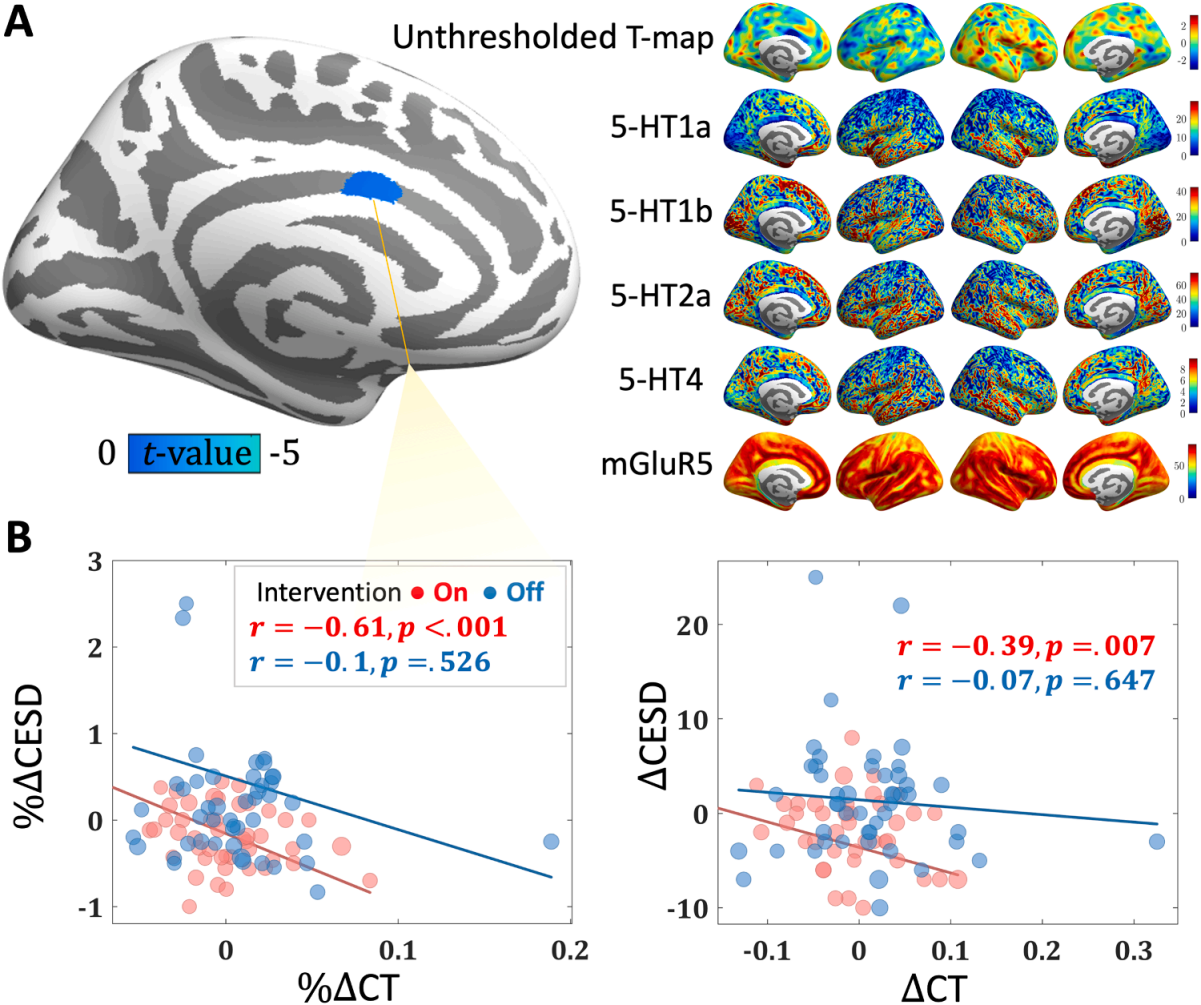
(A) Left: Cortical region showing a significant negative association between percent change in CES-D scores and cortical thickness (CT) during the intervention period (p<0.05, cluster-corrected). Right: Unthresholded T-map of the same association, presented alongside normative cortical maps of serotonergic (5-HT1A, 5-HT1B, 5-HT2A, 5-HT4) and glutamatergic (mGluR) receptor density. (B) Scatter plots illustrating the relationship between changes in CES-D scores and cortical thickness within the region identified in (A). Left: Percent changes in CES-D and CT; Right: Absolute changes (ΔCES-D and ΔCT), each plotted separately for the intervention (red) and non-intervention (blue) intervals.

During the active intervention periods, multiple regression analyses revealed a significant negative association between percent change in CES-D scores and cortical thickness in the left caudal ACC (peak MNI coordinate [–3, 6, 31]; peak t−value=−5.4; cluster size = 351 vertices; cluster-level corrected p<0.05; Fig. 3A). This indicates that greater reductions in depressive symptoms following the running intervention was associated with larger increases in cortical thickness in this region.

Moderation analysis confirmed the specificity of this association to the active intervention periods, as indicated by a significant interaction between symptom improvement and intervention status (p<0.05). No significant associations were observed during non-intervention intervals (see Fig. 3B). Complementing this finding, whole-brain vertex-wise analyses revealed no suprathreshold clusters during control periods, further supporting that brain–symptom coupling depends on active aerobic engagement.

Furthermore, vertex-wise spatial correlation analyses showed significant associations between the unthresholded t-map (depicting the relationship between changes in cortical thickness and CES-D scores during active intervention periods) and neurotransmitter distribution maps. Specifically, positive correlations were found with the spatial distributions of serotonergic and glutamatergic markers (Fig. 3A), including 5-HT1a ($r=0.12,\;\;p_{FDR}=0.047$), 5-HT1b ($r=0.08,\;\;p_{FDR}=0.027$), 5HT2a ($r=0.10,\;\;p_{FDR}=\;0.027$), 5HT4 ($r=0.10,\;\;p_{FDR}=\;0.027$), and mGluR5 ($r\;=\;0.12,\;\;p_{FDR}=0.027$). In contrast, no significant spatial colocalization was observed when the same analyses were conducted on cortical thickness–symptom associations during control intervention periods (i.e., when the exercise program was inactive), highlighting the specificity of the neurotransmitter–plasticity coupling to active aerobic intervention.

## Discussion

After seven 60-minute running sessions over two weeks, healthy young adults exhibited significant reductions in depressive symptoms. These symptom changes were accompanied by increased cortical thickness in the ACC, a region implicated in emotional regulation and cognitive control. Greater increases in ACC cortical thickness were associated with larger reductions in depressive symptoms during the active running intervention. Furthermore, significant spatial colocalization was observed between the symptom-thickness association map and the normative cortical distributions of several neurotransmitter receptors, including 5-HT1a, 5HT1b, 5HT2a, 5HT4, and mGluR5. These findings suggest that exercise-related changes in depressive symptoms covaried with ACC cortical thickness changes, and the spatial pattern corresponded with normative serotonergic and glutamatergic receptor distributions.

The observed reductions in depressive symptoms are consistent with prior evidence linking physical activity and exercise to more favorable mood-related outcomes (Noetel et al., 2024; Singh et al., 2023). A meta-analysis of 49 studies involving over 1.8 million participants reported that individuals with high physical activity levels exhibited a 17% lower risk of developing depression (Schuch et al., 2018). Exercise-related changes in mood may involve multiple psychological mechanisms, including reduced tension and anxiety and improved self-esteem and overall well-being (Markotic et al., 2020; Rebar et al., 2015). Nevertheless, because the present study included healthy young adults with subclinical depressive symptoms rather than patients with MDD, the observed changes should not be interpreted as evidence of antidepressant efficacy or clinical treatment effects.

Changes in depressive symptoms were also associated with changes in ACC cortical thickness. The ACC contributes to emotional regulation, cognitive control, and social functioning (Ruscheweyh et al., 2011), and structural abnormalities in this region have been reported in mood disorders, including depression and bipolar disorder (Drevets et al., 2008). Previous studies have demonstrated that exercise can induce structural changes in the ACC (Floel et al., 2010; Lin et al., 2020; McEwen et al., 2023; Tao et al., 2019), which correlate with reductions in depressive symptoms (Dotson et al., 2021; McLaren et al., 2016; Szymkowicz et al., 2016). The present findings extend this literature by showing covariation between ACC cortical thickness and depressive symptom changes during the running intervention. However, this association does not establish that cortical thickening mediated or drove the observed symptom reductions. Cortical thickness changes and symptom changes may instead represent parallel and potentially independent responses to exercise or may arise from shared physiological or psychological processes.

The unthresholded *t*-maps reflecting symptom–thickness associations showed significant spatial colocalization with the cortical distribution maps of serotonergic and glutamatergic neurotransmitter systems. This pattern is consistent with the established involvement of serotonergic and glutamatergic systems in depression-related processes (Erritzoe et al., 2023; Pahlavani, 2024) and with previous evidence linking physical exercise to alterations in central monoaminergic and glutamatergic signaling (Ji et al., 2017; Maddock et al., 2016; Melancon et al., 2012; Young, 2007). Former research have reported that exercise influences serotonergic activity, ACC-related neural function, and cortical glutamate concentrations in humans and animal models (Ohmatsu et al., 2014; Pahlavani, 2024; Zhou et al., 2021). However, the JuSpace analyses rely on normative receptor maps derived from independent samples and therefore provide only a neurobiologically informed context for the observed cortical thickness-symptom association. Future studies using molecular imaging approaches (e.g., PET or MRS) are warranted to directly assess exercise-related changes in neurotransmitter systems.

Several limitations should be considered when interpreting our findings. First, the sample size was relatively modest (n=48), which may have limited statistical power to detect subtle effects on cortical structure. Second, the study examined a brief two-week aerobic running intervention, and it remains unclear whether the observed effects generalize to other exercise modalities, intensities or durations. Moreover, the limited follow-up period precludes conclusions regarding the persistence of exercise-related changes in depressive symptoms and ACC cortical thickness, including whether these changes persist after exercise cessation or reflect transient short-term adaptations. Third, given the crossover design, potential carryover effects cannot be fully excluded. Exercise-related psychological or structural changes may persist into the subsequent control phase, potentially affecting its interpretation as a true baseline condition. Future studies with longer washout periods and follow-up assessments are needed to further characterize the persistence of these effects. Finally, we analyzed only the total CES-D score without examining individual symptom dimensions, limiting inferences about which depressive features are most responsive to aerobic exercise.

## Conclusion

In summary, a two-week aerobic exercise regimen, consisting of seven 60-minute running sessions, produced significant reductions in depressive symptoms among healthy young adults. These behavioral changes were accompanied by cortical thickness increases in the ACC, a region centrally involved in emotion regulation and cognitive control. Notably, the symptom-related cortical thickness changes were associated with the spatial distribution of key neurotransmitter receptors—specifically serotonergic (5-HT1a, 5-HT1b, 5-HT2a, 5-HT4) and glutamatergic (mGluR5) systems, providing neurochemical context for the observed exercise-related alterations. Collectively, these findings contribute to a better understanding of the neurobiological mechanisms underlying exercise-related mood changes in non-clinical populations and warrant further investigation in clinical populations.

## Statement

During the preparation of this work the authors used ChatGPT to improve the readability and language of the manuscript. After using these tools/services, the authors reviewed and edited the content as needed and take full responsibility for the content of the published article.

## Funding

This work was supported by the National Natural Science Foundation of China (Grant No. 62271415, 32360212, 61876156, 31900764).

## Contributions

Q.Y. Writing – original draft, Formal analysis, Data curation. Q.X Investigation, Formal analysis, Writing – review & editing. Y.L. Formal analysis, Data curation. Y.Y. Validation, Writing – review & editing. X.M. Investigation, Writing – review & editing. Z.Z. Formal analysis, Writing – review & editing. C. B. Writing – review & editing, Supervision. L.W. Investigation, Project administration, Writing - Review & Editing, Supervision, Funding acquisition. G.W. Conceptualization, Methodology, Visualization, Writing – review & editing, Supervision, Project administration, Funding acquisition.

## Declaration of competing interest

The authors declare that they have no conflict of interest.

## Data Availability

All data used in the present study were publicly available from the OpenNeuro database (https://openneuro.org/datasets/ds003799/versions/1.0.0).
